# A Holistic 4D Approach to Optimize Intrinsic and Extrinsic Factors Contributing to Variability in Microarray Biosensing in Glycomics

**DOI:** 10.3390/s23125362

**Published:** 2023-06-06

**Authors:** Paras H. Kundalia, Lucia Pažitná, Kristína Kianičková, Eduard Jáné, Lenka Lorencová, Jaroslav Katrlík

**Affiliations:** Department of Glycobiotechnology, Institute of Chemistry, Slovak Academy of Sciences, SK-84538 Bratislava, Slovakia; chempars@savba.sk (P.H.K.);

**Keywords:** microarray, biosensor, affinity-based methods, high-throughput glycoprofiling, glycan, lectin, non-contact spotting, piezo-electric dispensing

## Abstract

Protein–carbohydrate interactions happen to be a crucial facet of biology, discharging a myriad of functions. Microarrays have become a premier choice to discern the selectivity, sensitivity and breadth of these interactions in a high-throughput manner. The precise recognition of target glycan ligands among the plethora of others is central for any glycan-targeting probe being tested by microarray analyses. Ever since the introduction of the microarray as an elemental tool for high-throughput glycoprofiling, numerous distinct array platforms possessing different customizations and assemblies have been developed. Accompanying these customizations are various factors ushering variances across array platforms. In this primer, we investigate the influence of various extrinsic factors, namely printing parameters, incubation procedures, analyses and array storage conditions on the protein–carbohydrate interactions and evaluate these factors for the optimal performance of microarray glycomics analysis. We hereby propose a 4D approach (Design–Dispense–Detect–Deduce) to minimize the effect of these extrinsic factors on glycomics microarray analyses and thereby streamline cross-platform analyses and comparisons. This work will aid in optimizing microarray analyses for glycomics, minimize cross-platform disparities and bolster the further development of this technology.

## 1. Introduction

Glycosylation denotes one of the most characteristic features during the constant process of evolution in the majority of the fundamental units of life [1]. All secretory proteins and membrane proteins cotranslationally translocate the endoplasmic reticulum (ER)—Golgi machinery—where they are correctly folded, modified and subjected to quality control mechanisms. This also happens to be the premier site of glycosylation, making it the most copious and customary post-translational modification [2]. The template-independent biosynthesis of glycans grants them immense diversity and cumulatively impacts the appropriate folding, solubility, stability and function of proteins and lipids. Most of the eukaryotic cell surfaces, secreted molecules and extracellular matrices of multicellular organisms are decorated with complex glycoforms. Several of the above-mentioned rationales have led to the recurrent and consistent choice of glycans as the mediators between cells and the extracellular milieu, authorizing facile short-term and long-term adaptations to change environments and pathogen regimes [3]. In addition, these sugar chains (glycans) serve crucial structural roles in the cell walls of bacteria, archaea and fungi, resisting osmolar disparities between the cytoplasm and the extracellular environment and aiding in their pathogenicity [3,4]. Even budding viruses from infected cells acquire the glycosylation pattern from the host membranes [5].

The synthesis of glycoconjugates is a dynamic process, which is influenced by several parameters, such as the availability of donor sugar precursors, localization of enzymatic milieu, cell type and transient cellular signals [6]. Coherently, the secreted and cell-surface glycome serves as an intrinsic barometer of cellular wellbeing and thereby reflects the overall cellular status in health and disease [7]. It has also been well appreciated that glycosylation acts as a “universal self” in addition to protein and deoxyribonucleic acid (DNA) sequences and is used as a signature to discern “self” vs. “alien” [8]. The plethora of glycans and oligosaccharides that decorate the various glycoconjugates and polysaccharides potentially conceal extensive information, harboring a myriad of glycocodes, which need to be deciphered in biological and medical contexts. Healthy cells are known to avert immune recognition by virtue of their normal terminal glycosylation patterns. Therefore, alterations in cellular homeostasis manifested due to disease or stress precipitate as anomalies in glycosylation patterns, serving as sensitive and promising biomarkers [9]. Considering the universality of glycosylation, it becomes imperative to tap their patterns for a better comprehension of their functions.

**Figure 1:**
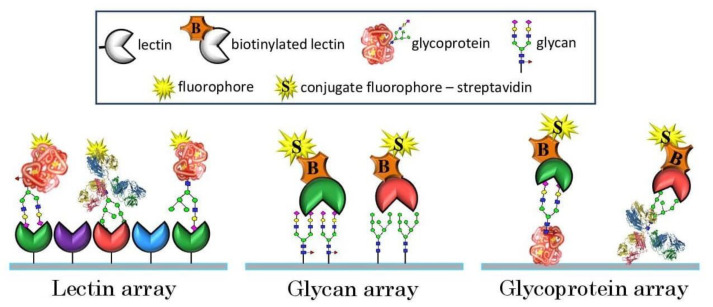
Illustration of biorecognition principle in lectin, glycan and glycoprotein arrays (from left to right).

The chemical construct of glycans as mixtures of different sugar forms confers them enormous heterogeneity in micro-, macro- and metadimensions [10]. Contemplating such 3M (micro-, macro- and metaheterogeneity) complexity of glycosylation, it is almost unfeasible to determine the complete glycoprofile of a given protein and to estimate the existing amounts of individual glycans. In this context, analytical tools ranging from one-dimensional, high-throughput glycoprofiling methods to approaches yielding an accurate structural elucidation of glycans from a sample are available. Most of these methods are principally based on chromatographic, electromigratory, optical or mass spectrometric approaches [11,12,13,14]. This implies (a) the detection and quantification of all types of glycans; (b) the segregation of individual isomers; and (c) the assignment of components to a particular structure, including the overall topology of the molecule and appropriate linkages. Practically, with the sophisticated techniques available at hand, this can still be pursued with limited sample numbers. Paradoxically, it is outlandish and inconceivable while dealing with tens and hundreds of samples where throughput is the prerequisite. Primarily, this is because the separation and identification of glycans from a sample or individual glycoprotein all at once is a tedious task since their numbers and types can be considerable. Moreover, in most of these studies, structural analysis becomes the sole objective, and the actual objective gets side-tracked. Therefore, it becomes extremely crucial to strategize, precisely define the research objective and assess the methods adopted, which will be time-efficient, cost-effective and yield the maximum information from the analysis of the sample. The premier step while strategizing glycoprofiling is to adopt the shortest route, which means eliminating procedures of excision and the mutual separation of glycans. For this purpose, affinity methods based on glycorecognition mediated by carbohydrate-binding proteins (CBPs) were proven as a suitable approach. CBPs are predominantly lectins, which are proteins of typically non-immune origin recognizing specific glycan motifs, i.e., particular monosaccharides linked in certain orientations, depending on individual lectin specificity. In general, lectin–glycan interactions are similar to antibody (Ab)–antigen (Ag) interactions, although barring the high specificity of Abs. Taking along the bioaffinity of CBP–glycan interactions, it was soon realized that this phenomenon can also be tested on a microarray platform in various formats (e.g., lectin, glycan and glycoprotein arrays) and it proved to be a game changer in achieving high-throughput for glycoprofiling, as indicated in Figure 1. The development of a microarray platform for glycan profiling emerged as a key elemental technology for achieving a high throughput, overcoming the above-mentioned hurdles and giving considerable information for deciding the subsequent approach to be adopted.

In contrast to purpose-specific studies that aim to interrogate individual parameters, such as linker effects or glycan density, the availability of studies encompassing crucial basic aspects of microarrays starting from microarray fabrication to data generation has been very limited. In this primer, our work comprehensively investigates the influence of various extrinsic factors, namely printing parameters, incubation procedures, printed array storage conditions and scanning parameters, on the CBP–carbohydrate interactions.

## 2. Factors Influencing Microarrays Analysis in Glycomics

**Figure 2:**
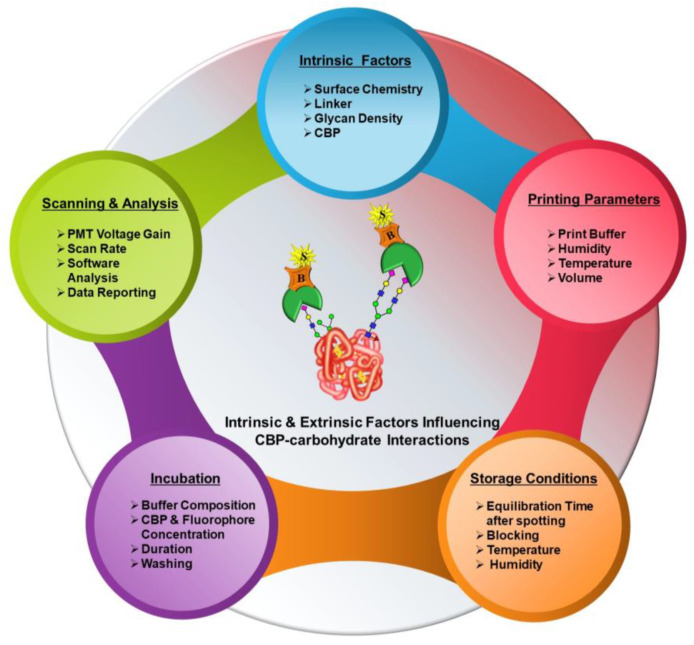
Schematic representation of intrinsic and extrinsic factors influencing microarray analysis of CBP–carbohydrate interactions. The color coding in the background from red to white (from top right to bottom left) indicates the extent of research work assessing these parameters.

The introduction of microarray technology since the beginning of the new millennium [15,16,17,18] at the frontiers of glycomics revolutionized the investigation of carbohydrate–protein interactions and the elucidation of their specificities in endogenous biological processes. Since then, microarrays, including glycan microarrays (glycoarrays), glycoprotein microarrays and lectin microarrays, have become customary analytical tools of choice to assess protein–carbohydrate interactions in a high-throughput manner [19]. With the progress in time, various sophistications have been made to perform targeted studies spanning the glycoprofiling of recombinant therapeutics [20], biomarker discovery [21,22], differential diagnosis [23] and many more. Realizing the time effectivity, minuscule sample consumption, throughput capacity and versatility of this tool, various distinct glycan, glycoprotein and lectin array formats [24] were assembled by customizing different components, such as slide-surface chemistries, linkers, glycan/lectin presentation, densities, printing technologies and conditions and so on. While all these studies principally aimed to interrogate protein–carbohydrate interactions, each of their respective customizations, from array fabrication to experimental analysis, scanning, data analysis and reporting led to several differences or inconsistencies in cross-platform comparison. Based on our understanding of the factors influencing glycoarray outcomes, they can be broadly classified into two categories, namely intrinsic and extrinsic. Figure 2 provides a comprehensive graphical overview of this.

### 2.1. Intrinsic Factors

Reflective of the terminology, these factors are intrinsic properties of the whole process of microarray analysis and can only be tweaked by using various combinations, but they cannot be altered. These factors include several components listed below.

#### 2.1.1. Slide-Surface Chemistry

The microarray slide-surface forms the substratum of this platform and hence is the most crucial intrinsic factor impacting the fabrication and overall outcome of the glycoarray analyses. In most cases, the silica surface is functionalized by various chemistries [25,26,27,28], which can complement the immobilization strategy [29,30] adopted to anchor the appropriate biomolecules either as biorecognition elements or analytes to be tested, which can be individual glycans, glycoproteins, neoglycoproteins, glycoconjugates or CBPs, such as lectins. It has been previously reported and established that the slide surface significantly influences the three-dimensional structure, surface capacity, background noise, ligand presentation, spot size and morphology of the arrayed molecules, ultimately affecting their interactions and reproducibility [31,32].

#### 2.1.2. Presentation and Linker Effects

Linker as the name suggests anchors the ligand to the molecular scaffold of the slide chemistry and thereby happens to be another important variable in the ligand presentation. Linker chemistry, length and orientation relative to surface chemistry appreciably influence ligand flexibility, ligand elevation above the surface, ligand presentation and analyte accessibility [32,33].

#### 2.1.3. Ligand Density

Depending on the molecular structure of the ligand, the density presented varies as per the type of ligand printed. This in turn affects the ligand recognition and binding by the analyte. Previous glycan array studies performed have indicated that monovalent interactions between glycan and a single lectin binding domain are typically weak. The multivalent presentation of carbohydrate ligands generally enhances the avidity of CBPs and effectuates tighter binding through the formation of multivalent complexes [34,35].

#### 2.1.4. Nature of the Analyte

Glycoarrays and glycoprotein microarrays are typically probed with CBPs and with CBP-containing samples, such as, e.g., viruses, whereas lectin microarrays are used for screening samples containing glycoproteins or glycans. Usually, the binding of a CBP on an array surface, apart from binding to its conventional binding partner, is accompanied by a multitude of interactions with the slide surface, linker and other adjacent lectin molecules. The extent of this influence principally is an intrinsic characteristic of the CBP depending on the morphology of its binding pocket, the molecularity and localization of these pockets, the localized surface charge and overall net charge and the steric accessibility of binding sites on a particular array. In addition, certain CBPs tend to exhibit lower background noise as against others [32,35].

### 2.2. Extrinsic Factors

Microarray fabrication and analyses entail microarray printing or dispensing; slide storage; experimental analyses or detection; scanning; and analysis. Each of these procedures comprises several components that are principally governed by external factors, such as the type of microarray spotter and spotting parameters calibrated, choice of buffers, scanning parameters, etc. None of these factors have intrinsic characteristics but rather are variable. Numerous studies probing the influence of specific aspects, such as different printing buffer compositions [28,36,37] and different humidity conditions during printing [37,38], on microarray analyses of the CBP–carbohydrate interactions have been performed. Additionally, cross-platform comparison studies collating glycan-binding profiles from different array platforms varying in several intrinsic and extrinsic factors and gauging the reliability of the results have also been reported [39,40]. Considering the multitude of factors involved with each step right from the preparation of the arrays to the end-point analysis, we propose and investigate additional factors that considerably impact the microarray outcomes of CBP–carbohydrate interactions. Basically, these are extrinsic factors, namely printing parameters, experimental procedures, printed slides storage, scanning and analysis (Figure 2). In this primer, the following work predominantly focuses on a systematic approach to design microarray formats and optimize these extrinsic factors, curtailing variability in the results of interaction analysis and devising an efficient and high-throughput microarray platform for glycoprofiling.

## 3. 4D Approach

**Figure 3:**
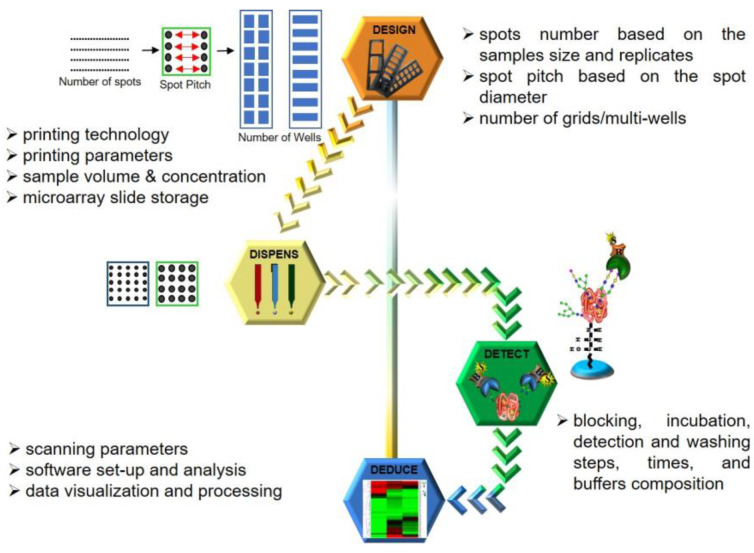
4D approach adopted to optimize factors contributing to variability in microarray analysis of CBP–carbohydrate interactions. The chronological order of the four major steps in optimization follows the solid arrow heads from Design to Dispense, Detect and Deduce.

Based on the microarray workflow, we hereby postulate a four-point strategy, which we term the 4D approach, derived from each of the stages strategized, namely Design, Dispense, Detect and Deduce, as illustrated in Figure 3. Parameters adopted for each stage are detailed in the Results and Discussion section.

### 3.1. Design: Designing the Format in Which the Array Must Be Printed

The primary rationale for using a microarray is to achieve the maximum analytical throughput for both the ligand and the analyte. Therefore, as per the number of ligands to be printed (spotted), a spotting grid must be designed to fit all the technical replicates of each ligand. A calculation and judgement have to be made to allow the spotting of the entire grid on the microarray slide in an appropriate multiwell format (e.g., 3, 4, 8, 16 or 32 wells), ultimately deciding the throughput and amount of an analyte that will be consumed.

### 3.2. Dispense: Calibration of Dispensing Parameter

Modern-day microarray spotters have generally switched from contact spotters to the non-contact mode of sample dispensing. Both these technologies have their respective pros and cons. However, irrespective of the printing technology, it is the nature of the sample or ligand to be printed that dictates the choice of print buffers being adopted. This, in turn, directs the choice of a buffer-compatible coating type of the printing nozzle, which along with sample chemistry is a major factor influencing the spot size and morphology. Pure samples are generally more homogenous when compared to complex mixtures and normally tend to distribute and immobilize uniformly over the microarray substrate. Hence, some samples can be dispensed at low volumes whereas others need to be dispensed in higher amounts. An increase in dispensing volumes indicates larger spots, which leads to larger spot diameters, decreasing the inter-spot distances (distances between the circumferences of two adjacent spots) while the inter-spot pitches (distances between the centers of two adjacent spots) remain constant. This effectively reduces the inter-spot distance, leaving marginal room for spotting anomalies/errors ultimately compromising throughput. Additionally, some samples or ligands based on their chemistry and viscosity give uniform spots at lower concentrations while others need to be spotted in higher amounts. The printing area humidity and temperature are other variables that influence sample drying after the spot has been dispensed thereby affecting spot morphology. Thus, calibrating these crucial printing parameters for individual sample/ligand types is a key aspect to obtain good spot morphology and attain optimized spotting. Once the slides have been printed, it also must be ensured that the unoccupied functional groups on the unspotted area are deactivated (blocked) and the slides must be stored under inert conditions.

### 3.3. Detect: Optimizing Experimental Procedure to Detect the Binding Interactions

Fine-tuning the dispensing parameters on the slide surface ensures efficient ligand immobilization and good spot morphology. However, this only secures the fabrication of the array, which is just the first half of the entire assay. After spotting the ligands on the slide, experimental procedures where the analyte actually interacts with the printed ligands need to be performed. This involves a sequence of incubation and washing steps, indicating the need for optimal buffer compositions to reconstitute the analyte, analyte concentration, incubation time and condition, and similar aspects for the fluorophore as well. In addition, the need to optimize the washing buffer composition and procedure goes unsaid. Once the slides are developed with a fluorophore, they need to be scanned at appropriate wavelengths using microarray scanners to detect the binding events. In the case of photomultiplier tube (PMT) detectors, the amount of voltage applied to the PMTs or scanning laser intensities is one of the key players influencing the readout of binding events. Moreover, the scanning rate or the speed at which the laser passes over the slide area is another aspect to be kept in mind. Accustomed laser power and detection gain that can especially tap the majority of the weak binding interactions without saturating the fluorescence signals generated by the stronger binding interactions become crucial facets of calibrating the detection of the binding events.

### 3.4. Deduce: Analyzing and Deducing the Nature of Interactions

Finally, the scanned images of the binding interactions need to be analyzed to be able to deduce and quantify analyzed binding events. Most of the microarray scanners are accompanied by analytical software that is basically able to estimate the difference in the fluorescence intensities of the spot where the binding interaction has occurred against the background. The software generally involves designing an analytical grid over the scanned image. Depending on the spotting, customizations such as specifying the spot size, pitch and area of the background to be considered during estimation are required to obtain the final readout. These analytical steps can also influence the signals and thereby need to be standardized. For analysis of our scans, individual grids were designed using Mapix software version 9.1.0 with a spot diameter ranging from 185–325 μm for 1–7 drop volumes. The inter-spot pitch was set at 450 μm with the following gridding properties: fixed diameter, maximum position offset (% pitch) = 30; photometric calculation—S/B border width (pixels) = 2; and background calculation—locally, background diameter (pitch) = 0.9.

## 4. Material and Methods

### 4.1. Materials

Phosphate-buffered saline (PBS) containing 0.01 M of phosphate buffer, 0.0027 M of KCl and 0.137 M of NaCl (pH 7.4 at 25 °C) and phosphate-buffered saline with Tween (PBST) containing PBS with 0.05% Tween 20, ethanolamine, boric acid, concentrated hydrochloric acid, human serum and fetuin lyophilized powder from bovine serum were purchased from Sigma-Aldrich (Saint-Louis, MO, USA). Bovine serum albumin, protease-free, was purchased from Lifecycle Biotechnologies (Cleburne, TX, USA). Blocking buffer Carbo-Free™ Blocking Solution (10× Concentrate) and biotinylated lectin Concanavalin A (ConA) were obtained from Vector Laboratories (Newark, CA, USA). Streptavidin conjugated with a fluorescent dye CF647 was from Biotium (Fremont, CA, USA).

### 4.2. Instrumentation

A sciFLEXARRAYER S1 microarray spotter and piezo dispense capillary PDC 90, type 3 were purchased from Scienion (Berlin, Germany). The microarray slides with epoxysilane coating NEXTERION^®^ E were bought from SCHOTT MINIFAB (Jena, Germany). An 8-well ProPlate^®^ multi-well chamber and ProPlate^®^ Multi-Array Slide System were obtained from Grace Bio-Labs (Bend, OR, USA). An InnoScan 710 fluorescence microarray scanner with Mapix software version 9.1.0 was purchased from Innopsys (Carbonne, France). A high-throughput wash station and microarray high-speed desktop centrifuge were bought from the Arrayit Corporation (Sunnyvale, CA, USA). A Multi Bio 3 programmable mini-shaker was obtained from Biosan (Riga, Latvia).

### 4.3. Incubation and Scanning Procedures

All the incubations were performed by attaching the slides to an 8-well ProPlate^®^ multi-well chamber. Every well was blocked with 250 μL/well of 1× Vector Carbo-Free™ Blocking Solution (diluted with deionized water) for one hour at RT. After this, the wells were washed 5 times with 300 μL/well of PBST to discard the excess blocking solution. Following this, all wells were incubated with 25 μg/mL of ConA solution (diluted in PBST) for one hour at RT with gentle rocking (8 rotation/minute). After this, the wells were again washed 5 times with 300 μL/well of PBST to discard the excess ligand. Then, the slides were incubated with 0.5 μg/mL (diluted in PBST) of streptavidin conjugated with a fluorescent dye CF647 for one hour at RT and gentle rocking. After the fluorophore incubation, the slides were washed 5 times with 300 μL/well of PBST to discard the excess fluorophore. Subsequently, the slides were detached from the 8-well gasket and the excess wash solution was decanted without completely drying the slides. Then, the slides were washed in the high-throughput wash station with deionised water for 5 min. At last, the slides were taken from the wash station and spin-dried with a microarray high-speed desktop centrifuge. After the slides were dried, they were first scanned with the Innopsys 710 scanner in preview mode. The laser power, which has two options, “low” and “high”, corresponding to 5 mW and 10 mW, was set to low for our scannings. Likewise, the detection gain (DG), i.e., the value for the gain percentage of the power applied to the photomultiplier tubes (PMTs) of the detector for each color channel, which can be set from 1–100, was set to 3, 5 and 8, which henceforth we term as DG3, DG5 and DG8, respectively. Every slide was scanned at DG3, DG5 and DG8, successively. Higher values of detection gain than DG8 led to the saturation of most of the signals from the printed samples. Since the samples had a higher dynamic range corresponding to our sample concentrations and volumes, we also scanned the arrays at the extended dynamic range (XDR) at low laser power, to obtain a signal range extension and avoid saturation. The XDR is a preset algorithm of the Innopsys scanner and has a dynamic range of >10^6^ arbitrary units (A.U.), making 20-bit images. Considering this, all the slides were then scanned at the XDR in accurate mode since the slide had a large number of spots with strong signals, with a scan speed of 5 μm/second to obtain the tiff images.

## 5. Results and Discussion

To perform this study, data were obtained by printing standard normal human serum and a standard glycoprotein fetuin, at five different concentrations ranging from 6.25–100 μg/mL and six different dispensing volumes ranging from 1 drop to 7 drops (∼480–520 picoliter/drop). Protease-free bovine serum albumin (BSA) was printed as a negative control as per the above combinations. Every combination was spotted in 10 technical replicates to accommodate >750 spots. An estimation was made to print slides in an 8-well format to occupy the maximum area per well. Every well was incubated with one lectin, Concanavalin A (Con A). We chose to use only one lectin as we predominantly wanted to test the influence of various extrinsic factors on the binding interactions for which one would serve this purpose. Con A was the lectin of choice primarily because of its specificity for branched and terminal mannose (High-Man, Manα-1,6 (Manα-1,3) Man). Both serum glycoproteins and fetuin are abundantly glycosylated with branched and terminal mannosylated glycans. A sufficient fluorescent signal was prerequisite of this study. Serum, being a complex sample, has a higher abundance of mannosylated glycans and gives a much higher signal as compared to fetuin. Moreover, it had been previously witnessed that Con A, when compared to other lectins, had performed well on different slide chemistries generating low, non-interfering background noise and expected ligand recognition, especially in the case of fetuin [32]. Considering the chronological sequence of extrinsic parameters coming into play and influencing the stages underlined in Section 4.3, we strategized our study to perform optimization in the following order.

### 5.1. Effect of Spotting and Scanning Conditions

**Figure 4:**
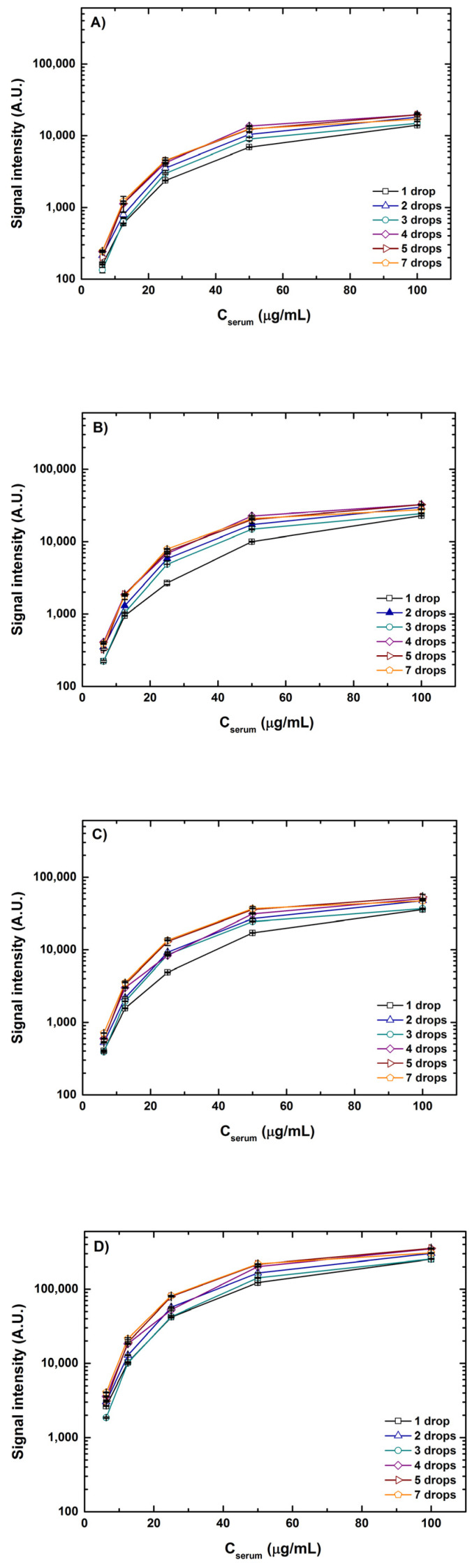
Fluorescence signal intensities for the interactions of lectin ConA with serum spotted in various concentration and volume combinations scanned at detection gains DG3 (**A**), DG5 (**B**) and DG8 (**C**) and in XDR mode (**D**), respectively. Error bars indicate standard error (*n* = 10).

As mentioned above with the various combinations of volumes and concentrations printed, it is principally the drop volume and not the drop concentration that was the deciding factor of spot size at 55% humidity and room temperature (20–25 °C). A marginal increase in spot diameter (~5%) was observed when the samples were spotted in concentrations in excess of 100 μg/mL. An increase in concentration from 6.25–100 μg/mL led to the formation of uniform spots curtailing anomalies and better spot morphology with spot diameters ranging from 185 to 325 μm. The influence of sample concentrations and drop volumes on the fluorescence intensities scanned at four different detection gain settings is depicted in Figure 4. For better apprehension of the results, signal intensities from interactions of lectin ConA with spotted serum samples of a 1–7 drop volume and a concentration of 6.25–100 μg/mL are plotted. A clear increase in individual spot intensities can be seen with the increasing detection gain (DG) in the order DG3 < DG5 < DG8 < XDR, which is very intuitive. Similar trends in signals were observed for all combinations of volumes and concentrations for both serum and fetuin. It is noteworthy that at detection gain of DG5 and DG8, many spots with higher volumes, say 4–7 drops, and higher concentrations of 50–100 μg/mL showed saturated signals. However, most of the saturation of signals resolved when the same spots were scanned at the XDR. For instance, the saturation value for scanning in standard mode (using manually set values of detection gain) is 65,535 A.U. and intensity values for a 100 μg/mL concentration of one drop and seven drop volumes at DG8 were ~37,000 A.U. (56% of the saturation value) and ~46,000 A.U. (70% of the saturation value), respectively. However, the range of signal intensity values for scanning in XDR mode is >10^6^ A.U. and signal intensity values for the same combination were ~250,000 A.U. (only 25% of the saturation value) and ~300,000 A.U. (30% of the saturation value), respectively. XDR scanning could capture a wider range of signals without saturating the intensities and thereby giving a better view of both weak and strong interactions detected simultaneously.

**Figure 5:**
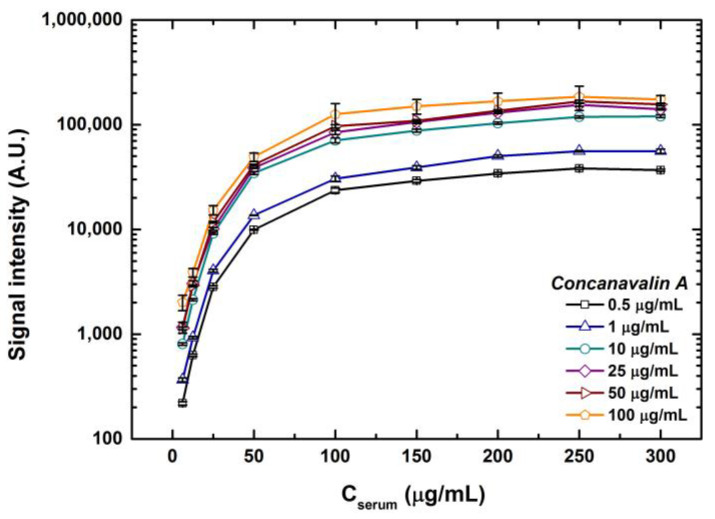
Fluorescence signal intensities for the interactions of 6 different lectin ConA concentrations with serum spotted in various concentrations scanned in XDR mode. Error bars indicate standard error (*n* = 10).

Looking at the graphs from Figure 4, it is also evident that with the increase in concentration for each volume combination the rise in signal intensity gradually skews, generating a saturation curve. This instigates a curiosity regarding what will be the saturation point or what can be the maximum signal where no further rise in fluorescence intensity can be seen. To test this, we printed an additional five concentrations higher than 100 μg/mL of serum and fetuin reaching a maximum of 3 times the highest concentration spotted for the other tests. Inspired by the range of lectin concentrations adopted by previous cross-platform studies [39,40], we also tested a range of lectin concentrations, 0.1, 0.3, 0.5, 1, 10, 25, 50 and 100 μg/mL of ConA. The signals obtained from the two lowest concentrations of lectins were very low and were not fit to be analyzed. This is because the ligands spotted in our case, although abundantly glycosylated, are complex matrixes and not pure glycans. Thus, a certain threshold for lectin concentration is essential, below which the lectins interact with the glycans feebly giving poor signals. For better interpretation, intensities of a 1-drop volume of spotted serum with all the concentrations are depicted in Figure 5.

Comparing the lectin concentrations, the highest rise in the intensities was observed for lectin concentrations from 0.5 to 10 μg/mL, which was an approximately 60–100% rise in intensities for the entire range of sample concentrations. Similar trends were observed for fetuin. In fact, serum concentrations above 250 μg/mL for a 1-drop volume showed a marginal dip in fluorescence intensities. Therefore, it can be deduced that a ConA lectin concentration up to 10 μg/mL was essential to occupy most of the mannosylated glycans in the serum. Further increases in lectin concentration had a marginal effect on the rise in signal intensities, indicating that a ConA concentration range from 0.5 to 10 μg/mL should be a good choice to glycoprofile serum samples. Undoubtedly, this can vary from sample to sample.

### 5.2. Incubation Steps

#### 5.2.1. Effect of Blocking Buffer

A wide range of different blocking buffer compositions for different slide-surface chemistries are available in the literature [28,32,37,38,39,40]. Considering the epoxide-coated slide adopted for our work, we tested eight different buffer combinations mentioned in Table 1. We tried buffer combinations of water and BSA in the presence and absence of surfactant. Similarly, these variations were also tried with phosphate-buffered saline solution and ethanolamine. We also tested the commercial blocking buffer, i.e., Carbo-Free™ blocking solution. The reason to test these buffer combinations was to decrease the background noise, which could mask the signals from weak interactions, as low as possible. Therefore, we wanted to find the blocking buffer that would efficiently deactivate all the reactive functional groups after printing, obtaining the least background noise, providing a considerable signal-to-noise ratio (SNR) and suiting the lectin to establish an efficient binding interaction. It had been reported previously that many lectins require some ions as cofactors to interact with their ligands [32].

**Figure 6:**
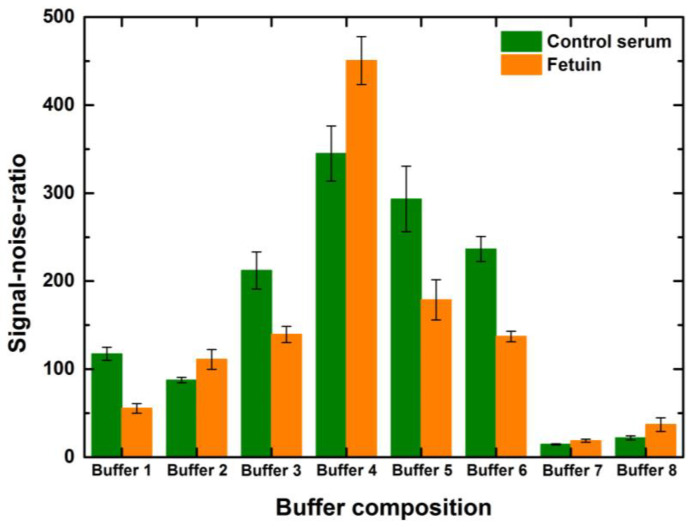
The effect of blocking buffer composition on signal-to-noise-ratio values of the interaction of lectin ConA with serum and fetuin printed on the microarray slide (1-drop volume, 50 μg/mL). Error bars indicate standard error (*n* = 10). The microarray slides were blocked with the eight buffers described in Table 1.

It can be observed that the rise in the SNR was more than 100% for the given concentrations of serum and fetuin for buffers 3, 4, 5 and 6 when compared to the SNR values of other buffers for the same concentration and volume combination of the spot (Figure 6). Generally, the serum spots might show higher SNR values than fetuin spots due to the higher extent of mannosylation of serum proteins compared to fetuin and correspondingly higher signal intensities. However, localized background deviations and the calculations of background noise significantly influence the individual spot SNR values. This pressingly indicates the need for efficient blocking of the array and uniform background noise. In addition, here we look holistically at the overall performance of the blocking buffer undermining the individual spot inconsistencies to judge the blocking buffer performance. The performance of different blocking buffer combinations was more or less alike to the above-mentioned trends for other combinations of concentrations and drop volumes. Thus, PBS with 0.05%Tween and 3% BSA was a good buffer composition for epoxide slides and the ConA lectin used for the incubation. Despite the SNR being higher for buffer 4, we chose to continue further blocking all the other assays with the commercial variant primarily to minimize batch-to-batch variations, which would have been higher in the case of our in-house buffer 4.

#### 5.2.2. Effect of Blocking Time

**Figure 7:**
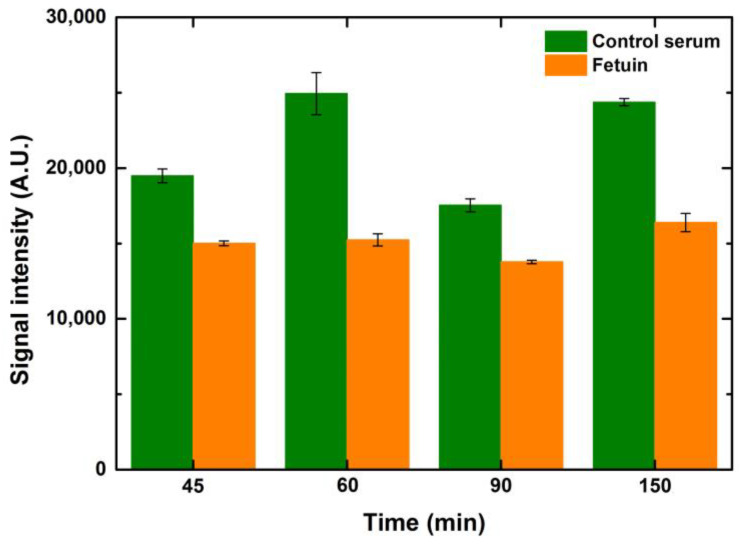
The effect of blocking time on the signal intensities of the interactions of lectin ConA with serum and fetuin printed on the microarray slide (1-drop volume, 50 μg/mL). Error bars indicate standard error (*n* = 10).

Blocking times from 45–150 min were tested to see the effect on signal intensity and background noise. While testing these variations, the incubation times of lectin and fluorophore were kept constant at 1 h each. Intensity values did not change much over 60 min of blocking for any of the ranges of concentrations and volumes. However, extended blocking times over one hour increased the background noise, reducing the SNR values. Since the composition of the commercial buffer is unknown, it is not possible to comment on the underlying reasons for such an observation. Intensity values of 1-drop volume for different blocking time points are depicted in Figure 7.

#### 5.2.3. Effect of Lectin Incubation Time

**Figure 8:**
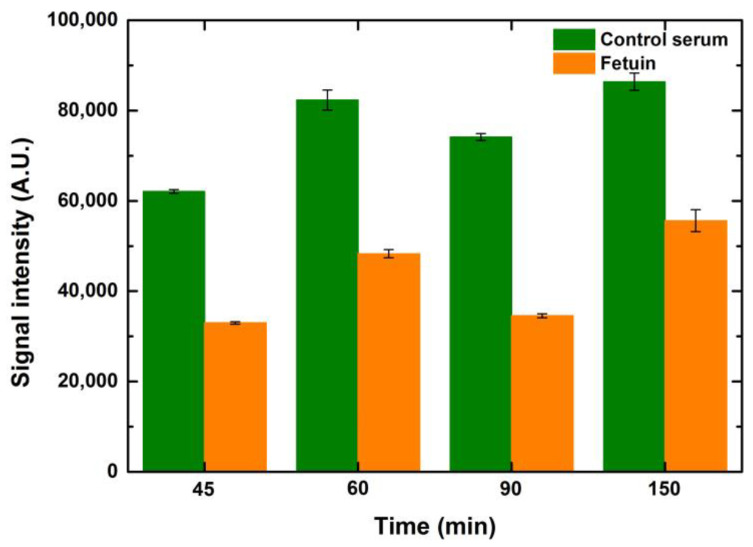
The effect of lectin incubation times on the signal intensities of the interaction of lectin ConA with serum (green bars) and fetuin (orange bars) printed on the microarray slide (1-drop volume, 100 μg/mL). Error bars indicate standard error (*n* = 10).

Similar to the optimization of the blocking time, ConA lectin (25 μg/mL) was also incubated at time points ranging from 45 to 150 min to observe the effect of lectin incubation times. It is evident that the signals were better when the incubation time increased by 15 min from 45 to 60 min for control serum and fetuin. However, any further lengthening of lectin incubation did not help to improve the signals further, as depicted in Figure 8. This can be attributed to the occupancy of the majority of the mannosylated glycans by ConA in an hour of incubation. Hence, there were negligible ligands left for ConA to bind after one hour of incubation. While performing these variations in lectin incubation times, the blocking and fluorophore incubation times were kept constant at one hour each. Increases in drop volume for both serum and fetuin depicted a relative increase in signal intensity when compared to the preceding drop volume, for the respective concentrations of both serum and fetuin, at all time points. The overall trend, over the tested time points, was similar for the entire range of drop volumes and concentrations for both serum and fetuin.

#### 5.2.4. Effect of Fluorophore—Streptavidin Incubation Time

**Figure 9:**
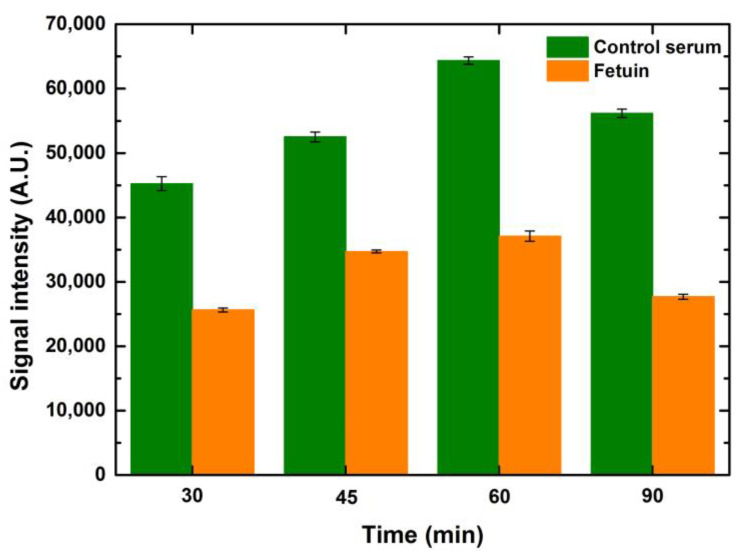
The effect of dye incubation times on signal intensities of the interaction of lectin ConA with serum (green bars and fetuin (orange bars) printed on microarray slide (1-drop volume, 100 μg/mL). Error bars indicate standard error (*n* = 10).

Taking the incubation timing optimization study further, we also tested the incubation timing of the fluorophore, which in our case was streptavidin conjugated with CF 647 at four different time points, namely 30, 45, 60 and 90 min, keeping the blocking and lectin incubation time constant at one hour. An overall increase in signals was observed when the fluorophore incubation time increased. The signals peaked at one-hour incubation of the fluorophore for both serum and fetuin. Looking at the increase in the binding of the analyte over a similar range of time points, it can be anticipated that the fluorophore incubation will also demonstrate a similar trend as the fluorophore binds to the analyte. Thus, the more the analyte is bound to the ligand, the higher the amount of fluorophore that will bind to the analyte and generate higher signals, as shown in Figure 9. The gradual dip in intensity observed during the extended incubation time over an hour can be seen as a reflection of the scarcity of analyte available for binding by the fluorophore. Higher drop volumes for serum and fetuin showed a relative rise in signal intensity when compared to the preceding drop volume for the respective concentrations of both serum and fetuin, at all time points. The overall trend, over the tested time points, was similar for the entire range of drop volumes and concentrations for both serum and fetuin.

### 5.3. Slide Storage Conditions

Slide-surface chemistries can possess very reactive surfaces, as in the case of N-hydroxysuccinimide (NHS)-coated slides and very stable surface chemistries, such as streptavidin-coated slides. The effect of prolonged exposure times of reactive surfaces, such as NHS, to near ambient conditions while printing on the microarray results has been tested previously [37]. A decrease in CBP binding has been observed due to the hydrolysis of NHS esters after 36 h of printing. Taking this further, we tried to investigate the effect of different storage conditions on CBP binding signals. We tested four different storage conditions after printing and equilibrating the slides at 65% humidity for 8 h so that all the ligand molecules bind to the surface efficiently, ensuring complete immobilization. The four different storage conditions chosen are as follows: (i) blocking and lectin incubation immediately after equilibration; (ii) blocking and storing the slides at −20 °C by vacuum sealing after equilibration and performing lectin incubation after 24 h of storage; (iii) storing the slides at −20 °C by vacuum sealing, without blocking after equilibration and performing lectin incubation after 24 h of storage; (iv) storing the slides at 4 °C without blocking and without vacuum sealing after equilibration and performing lectin incubation after 24 h of storage.

**Figure 10:**
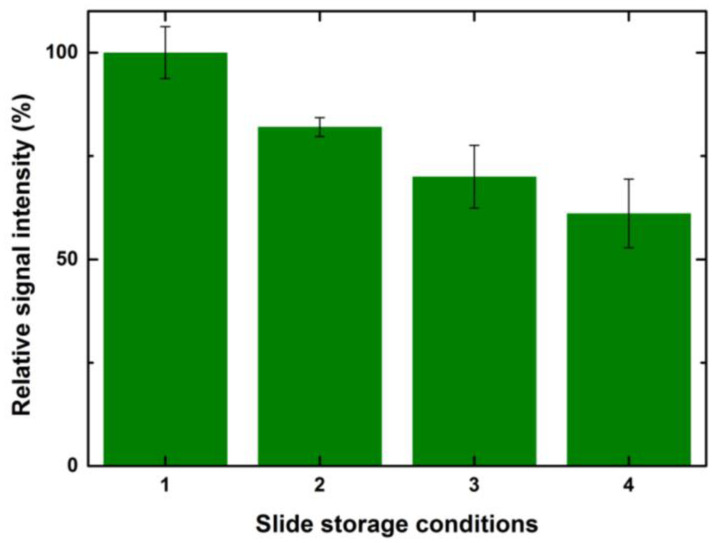
Relative intensity values of interaction of lectin ConA with serum printed on microarray slide (-drop volume, 100 μg/mL) at storage conditions (1) after 8 h at 25 °C; (2) after 24 h at −20 °C, blocked and sealed; (3) after 24 h at −20 °C, sealed without blocking; (4) after 24 h at 4 °C, without blocking or vacuum sealing. Error bars indicate standard error (*n* = 10).

A general trend of decrease in binding signals was observed with prolonged storage times, exposure to humidity and an increase in temperature. To elaborate further, slides that were immediately incubated with lectin after equilibration showed the highest signals overall for all tested concentrations and drop volumes when compared to other storage conditions. The second in the order were slides that were blocked, vacuum sealed, stored at −20 °C and incubated after 24 h. A further decrease in the signals was observed for unblocked slides despite storing at −20 °C. Microarray slides that were stored at 4 °C and incubated one day later showed the lowest signal. Figure 10 shows a relative percentage decrease in signal intensities for the above-mentioned storage conditions for a spotted 1 drop of control serum at 100 μg/mL.

Considering the signals obtained after 8 h of incubation as 100%, the relative drop in the intensities for the second condition was ~20%. There was a further 10% decrease in the signals for the arrays stored at −20 °C without blocking. The arrays stored in the refrigerator at 4 °C without blocking had 60% signal values when compared to the slides that were incubated after printing. A similar trend with values varying +/− 5% was observed for other drop volumes and concentrations. TheeExposure of reactive epoxide functional groups on the unspotted areas of the slide to a higher humidity and room temperature led to the formation of water-soluble diols that might bleach from the surface and also lead to the loss of spotted ligand [41].

### 5.4. Cumulative Understanding

Summarizing all the above optimizations, it would be appropriate to infer that extrinsic factors, namely printing parameters, buffer compositions, storage conditions, incubation parameters and scanning parameters, which were previously undermined and presumed to be impassive [35], rather considerably affect the microarray outcomes obtained from binding interactions. Several studies report the scan images of incubated microarray slides wherein spots with varying morphology and diameters can be observed [37,40]. Keeping aside the spot aesthetics, it generates an ambiguity regarding the printing parameters, such as the drop volumes and the concentrations that will be adopted to fabricate the arrays. Additionally, uniform spot morphology becomes extremely pivotal for setting the grid for analysis in most of the microarray analytical software. Hence, uniform spot morphology, as well as printing, will steer efficient analysis, abiding by the minimum information about a microarray experiment (MIAME) guideline [42], obtain reliable results and minimize cross-platform variances. It will also enhance the possibility of coupling microarrays with other platforms [43], augmenting the scope of this platform [44,45,46]. Apart from printing parameters, most of the microarray platforms developed utilize fluorescence for the detection of binding events. Therefore, methods to scan the slides and acquire and process microarray images, aligning the grid and most importantly judging spotting anomalies while analyzing, are fundamental to procure quality data. Endeavors to optimize printing microarrays [47], such as the experimentation above, and process microarray data [48] will aid in the microarray analyses for beginners and experts, paving the way for obtaining uniform results and further enhancement of this platform.

Having said this, further experimentation with different types of samples and analytes, along with assessments of other parameters, such as temperature, incubation steps, imaging and analysis, need to be performed. This could also be indicated as a shortcoming of this study, the scope of which is endless. In this primer, the 4D approach will help to cater microarray analysis and obtain reliable results.

## 6. Conclusions

Microarray analyses have become a preeminent tool for high-throughput evaluation in glycomics research, including the study of CBPs binding properties, mining glyco-biomarkers of various pathological conditions and comprehending immune responses and quality control regimes. They are crucial for determining the physiological ligands of CBPs, allowing the swift hypothesizing of glycan-binding analytes and deciding the further strategy to validate these ligands using other approaches. In addition, they serves as an excellent preliminary assessment tool to hypothesize and determine the binding specificities circumventing the immanent biases of other techniques, such as surface plasmon resonance (SPR), frontal affinity chromatography (FAC) and isothermal calorimetry (ITC), where prior background about CBP specificity is prerequisite. While different array platforms bespeak consensus about these binding interactions, they lack cross-platform consistency, especially in the case of moderate and weak binding interactions. This work highlights several other extrinsic factors, such as printing parameters, including drop volume and/or sample concentration, blocking buffer composition, incubation procedures, scanner detection gains and data analyses, impacting the microarray outcomes of CBP–carbohydrate binding interactions. Drop volumes and to a lesser extent sample concentrations along with print buffer composition and humidity are the most crucial factors influencing spot size and morphology. Testing the range of concentrations to be printed as well as the concentration of the analyte will help to maximize the signal intensities, especially for weak interactions, and optimize detector gains. Printed slides must be incubated after equilibration and utilized at the earliest time possible, to obtain the best results. Likewise, spotted arrays must be blocked efficiently, vacuum sealed and stored at −20 °C in completely dry conditions to avoid a loss in the signal intensities of the binding interactions. Blocking buffer compositions depending on the slide chemistry and type of the analyte must be tested to maximize the SNR and minimize background noise. The incubation procedure, especially incubation times, must be titrated to attain the complete binding of the ligand and the analyte, especially in the case of weak interactions. Scanner detection gains, depending on the scanner type, must be tested at different voltages applied to the PMTs to obtain adequate signal intensities from weak binding interactions without saturating the stronger interactions that generate pronounced signal intensities.

Optimizing the factors contributing to variability can help to achieve more accurate results and determine the most probable binding partners. This can further aid in determining binding kinetics and untangling higher-order questions about glycan binding. The 4D approach will assist in fabrication and microarray analyses and the further development of this tool will enhance its utility.

## Figures and Tables

**Table 1:** Buffer compositions adopted.

Buffer Number	Buffer Composition	pH
Buffer 1	H_2_O + 3% BSA	7.03
Buffer 2	H_2_O + 0.05% T + 3% BSA	7.03
Buffer 3	PBS + 3% BSA	7.4
Buffer 4	PBS + 0.05%T + 3% BSA	7.4
Buffer 5	1x Carbo-Free™ blocking solution	8.2
Buffer 6	ETA	adjusted to 8
Buffer 7	ETA + 3% BSA	adjusted to 8
Buffer 8	ETA + 0.05% T + 3% BSA	adjusted to 8

BSA—bovine serum albumin; T—Tween 20 (T); PBS—phosphate-buffered saline; ETA—ethanolamine. pH of buffers 6–8 was adjusted by adding concentrated hydrochloric acid.

## Data Availability

The datasets generated and/or analyzed during the current study are available from the corresponding authors, on reasonable request.
